# Structures of trehalose synthase from *Deinococcus radiodurans* reveal that a closed conformation is involved in catalysis of the intramolecular isomerization

**DOI:** 10.1107/S1399004714022500

**Published:** 2014-11-22

**Authors:** Yung-Lin Wang, Sih-Yao Chow, Yi-Ting Lin, Yu-Chiao Hsieh, Guan-Chiun Lee, Shwu-Huey Liaw

**Affiliations:** a Institute of Biochemistry and Molecular Biology, National Yang-Ming University, Taipei 11221, Taiwan; b Department of Life Sciences and Institute of Genome Sciences, National Yang-Ming University, Taipei 11221, Taiwan; c Department of Life Sciences, National Taiwan Normal University, Taipei 11677, Taiwan; d Department of Medical Research and Education, Taipei Veterans General Hospital, Taipei 11217, Taiwan

**Keywords:** trehalose synthase, intramolecular isomerization, glycoside hydrolase family 13, conformational change, enzyme mechanism

## Abstract

Trehalose synthase catalyzes the simple conversion of the inexpensive maltose into trehalose with a side reaction of hydrolysis. Here, the crystal structures of the wild type and the N253A mutant of *Deinococcus radiodurans* trehalose synthase (DrTS) in complex with the inhibitor Tris are reported. DrTS consists of a catalytic (β/α)_8_ barrel, subdomain B, a C-terminal β domain and two TS-unique subdomains (S7 and S8). The C-terminal domain and S8 contribute the majority of the dimeric interface. DrTS shares high structural homology with sucrose hydrolase, amylosucrase and sucrose isomerase in complex with sucrose, in particular a virtually identical active-site architecture and a similar substrate-induced rotation of subdomain B. The inhibitor Tris was bound and mimics a sugar at the −1 subsite. A maltose was modelled into the active site, and subsequent mutational analysis suggested that Tyr213, Glu320 and Glu324 are essential within the +1 subsite for the TS activity. In addition, the interaction networks between subdomains B and S7 seal the active-site entrance. Disruption of such networks through the replacement of Arg148 and Asn253 with alanine resulted in a decrease in isomerase activity by 8–9-fold and an increased hydrolase activity by 1.5–1.8-fold. The N253A structure showed a small pore created for water entry. Therefore, our DrTS-Tris may represent a substrate-induced closed conformation that will facilitate intramolecular isomerization and minimize disaccharide hydrolysis.

## Abstract 2

Crystal structures of the wild type and the N253A mutant of trehalose synthase from *D. radiodurans* in complex with the inhibitor Tris have been determined at 2.7 and 2.21 Å resolution, respectively, and they display a closed conformation for catalysis of the intramolecular isomerization.

## 1. Introduction   

Trehalose is a nonreducing disaccharide formed by an α,α-1,1-linkage of two glucose molecules and displays exceptional stability. This sugar is used by a wide variety of organisms for a variety of key functions such as as an energy/carbon source, for signalling and for protein/membrane protection, as well as being used as a bacterial cell-wall component (Elbein *et al.*, 2003 ▶). Trehalose plays an essential role in *Mycobacterium tuberculosis* metabolism and thus enzymes involved in the biosynthesis of this sugar serve as possible drug targets (Hunter *et al.*, 2006 ▶). In addition, trehalose stabilizes a wide variety of biomolecules and hence has been used widely in the food, cosmetics, medical and biotechnological industries. Trehalose synthase (TS) catalyzes the reversible conversion of the inexpensive maltose into trehalose in the absence of any coenzyme. This enzymatic synthesis has a number of advantages, namely a simple reaction, high substrate specificity and low cost; hence, this could easily form a potential method for the production of trehalose.

TS belongs to glycoside hydrolase family 13 (GH13), which includes a diverse range of carbohydrate-metabolizing enzymes and has been further divided into 35–37 subfamilies (Stam *et al.*, 2006 ▶; Cantarel *et al.*, 2009 ▶). The GH13 enzymes share a catalytic (β/α)_8_ barrel as their structurally conserved core together with a few conserved regions; this is despite their having a relatively low overall sequence identity (Svensson, 1988 ▶). They also include a variable number of member-unique subdomains that as yet remain without any known function. As for other GH13 enzymes, mechanistic analysis of TS from *M. smegmatis* (MsTS) demonstrated that this enzyme employs a double-displacement mechanism with a covalent glycosyl-enzyme intermediate (Zhang *et al.*, 2011 ▶). Further kinetic studies have suggested an intramolecular mechanism within an enclosed active site and that the conformational changes of the protein are rate-limiting. However, the recently published structures of MsTS and *M. tuberculosis* TS (MtTS) are nearly identical and show an inactive open conformation (Caner *et al.*, 2013 ▶; Roy *et al.*, 2013 ▶). In the present study, we have determined crystal structures of the wild type and the N253A mutant of TS from *Deinococcus radiodurans* (DrTS) that reveal a closed conformation for intramolecular isomerization. The available TS structures demonstrate that the rate-determining conformational changes are mediated mainly by TS-unique insertions that are involved in modulating the opening and closing of the active site. Such conformational changes that involve the intervention of member-unique subdomains during enzyme catalysis can also be observed in some other GH13 members.

## 2. Materials and methods   

### 2.1. Protein preparation and sedimentation analysis   

Mutational analysis was carried out using a QuikChange site-directed mutagenesis kit (Stratagene). The recombinant wild-type and mutant DrTS were expressed in *Escherichia coli* BL21 (DE3) cells using the pET-23a(+) vector (Qiagen; Wang *et al.*, 2007 ▶). Luria broth cultures were grown at 310 K and induced with 0.4 m*M* isopropyl β-d-1-thiogalactopyranoside at 289 K for 16 h. Cell pellets were resuspended in lysis buffer consisting of 20 m*M* sodium phosphate, 500 m*M* NaCl pH 7.4 and lysed using a French press. After the removal of cellular debris by centrifugation at 39 000*g* at 277 K for 30 min, the crude extract was applied onto a 5 ml nickel–nitrilotriacetic acid column (Qiagen). After washing with 20–60 m*M* imidazole, the protein was eluted with 250 m*M* imidazole and dialyzed against 20 m*M* HEPES pH 7.5, 100 m*M* NaCl, 1 m*M* dithiothreitol at 277 K. The molecular mass in solution was estimated using a Beckman-Coulter XL-A analytical ultracentrifuge with an An50Ti rotor. Sedimentation-velocity centrifugation was performed at 293 K and 42 000 rev min^−1^ with double-sector epon charcoal-filled centrepieces. The absorption of the cells at 280 nm was scanned every 5 min for 5 h and the data were fitted to a continuous *c*(*s*) distribution model using *SEDFIT* (Schuck *et al.*, 2002 ▶). All size and shape distributions were analyzed at a confidence level of *p* = 0.95 by maximal entropy regularization and a resolution *N* of 200 with sedimentation coefficients between 0 and 20 S.

### 2.2. Activity assay   

The isomerase and hydrolase activities of DrTS were determined by measuring the amount of trehalose and glucose produced from maltose, respectively (Wang *et al.*, 2007 ▶). The inhibitory effect of Tris on the DrTS isomerase activity was determined in an assay solution by the addition of 10 µg purified DrTS to 100 µl 50 m*M* maltose solution in 20 m*M* sodium phosphate pH 7.4 at 20°C for 2 h. For the mutants, the TS activity was assayed in a reaction mixture consisting of 200 µl 0.25 mg ml^−1^ purified DrTS and 800 µl 125 m*M* maltose solution in 20 m*M* sodium phosphate pH 7.4 at 20°C for 2 h. The activity assay for each mutant was carried out in quadruplicate. The reaction was terminated by heating the mixture in boiling water for 15 min. The amount of maltose, trehalose and glucose in each reaction mixture was measured using a high-performance liquid-chromatography system (Schambeck SFD 2100) equipped with a refractive-index detector (SFD, RI 2000) at a flow rate of 1 ml min^−1^. A carbohydrate-analysis column (6.0 × 150 mm, Shodex SZ5532) equilibrated with 75% acetonitrile, 24% Milli-Q water and 1% formic acid was used. One unit of the isomerase or hydrolase activity was defined as the amount of enzyme that catalyzes the formation of 1 nmol of trehalose or glucose per minute.

### 2.3. Structure analysis   

The initial crystallization screening was performed with screening kits using the hanging-drop vapour-diffusion method at 288 K. The hanging drops were mixtures of 2 µl reservoir solution and 2 µl protein solution. Crystals of the wild-type protein were grown in 9% PEG 4000, 0.2 *M* sodium acetate trihydrate, 0.3 *M* Tris–HCl pH 8.5 using a protein solution at 30 mg ml^−1^ in 6–8 weeks. The N253A mutant crystals were obtained in 11% PEG 4000, 0.2 *M* sodium acetate trihydrate, 0.3 *M* Tris–HCl pH 8.5, 5% glycerol using a protein solution at 60 mg ml^−1^ in two weeks. X-ray diffraction data were collected and processed on beamlines BL13B1 and BL15A1 at NSRRC, Hsinchu, Taiwan. The wild-type and N253A crystals belonged to space groups *P*2_1_ and *P*2_1_2_1_2_1_, respectively. The structures were determined by the molecular-replacement method using the MsTS structure as a template (Caner *et al.*, 2013 ▶); the search models were generated by *MODELLER* (Šali & Blundell, 1993 ▶) and the phase problem was solved using *Phaser* (McCoy *et al.*, 2007 ▶). Further refinement was performed with *Coot* (Emsley *et al.*, 2010 ▶) and *REFMAC*5 (Murshudov *et al.*, 2011 ▶), and the Ramachandran analysis was performed using *MolProbity* (Chen *et al.*, 2010 ▶). Noncrystallographic symmetry restraints were applied in structural refinement of the wild type but not in that of the N253A mutant. The statistics of data collection and refinement are summarized in Table 1 ▶. Figures were generated using *PyMOL* (DeLano, 2002 ▶).

## 3. Results and discussion   

### 3.1. The DrTS structure   

**Figure 1:**
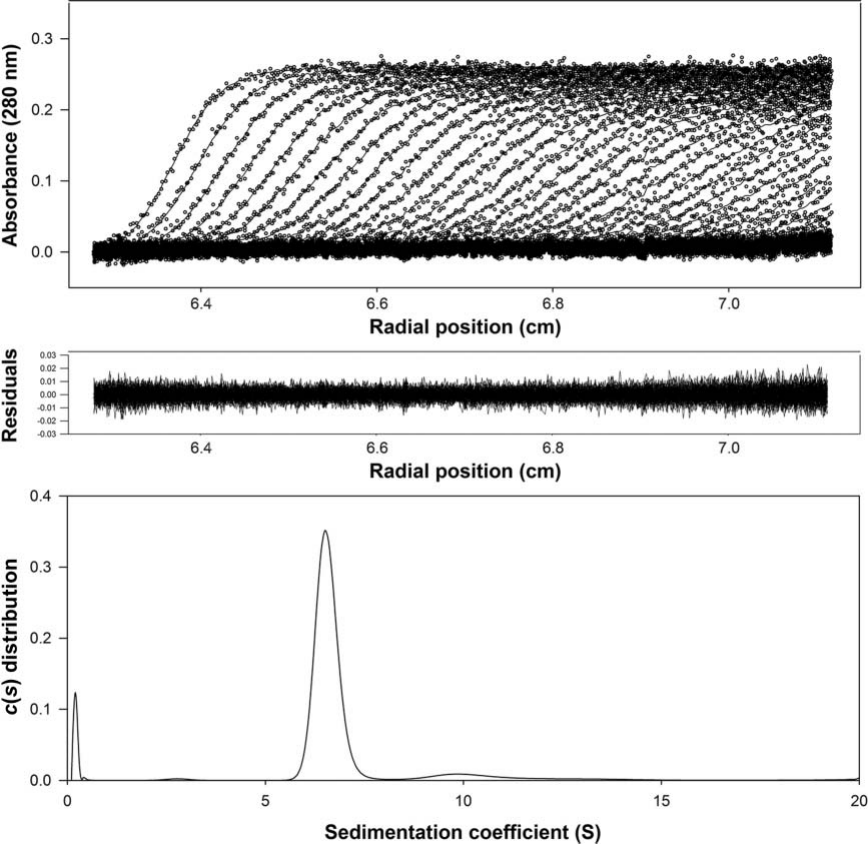
Sedimentation-velocity analysis of DrTS. The enzyme concentration was 0.1 mg ml^−1^ in 20 m*M* Tris–HCl pH 7.5, 100 m*M* NaCl. The three panels represent the optical traces (top), the residuals of the model fitting (middle) and the sedimentation-coefficient distribution (bottom). The ultracentrifugation analysis demonstrated that DrTS exists as a dimer in solution.

**Figure 2:**
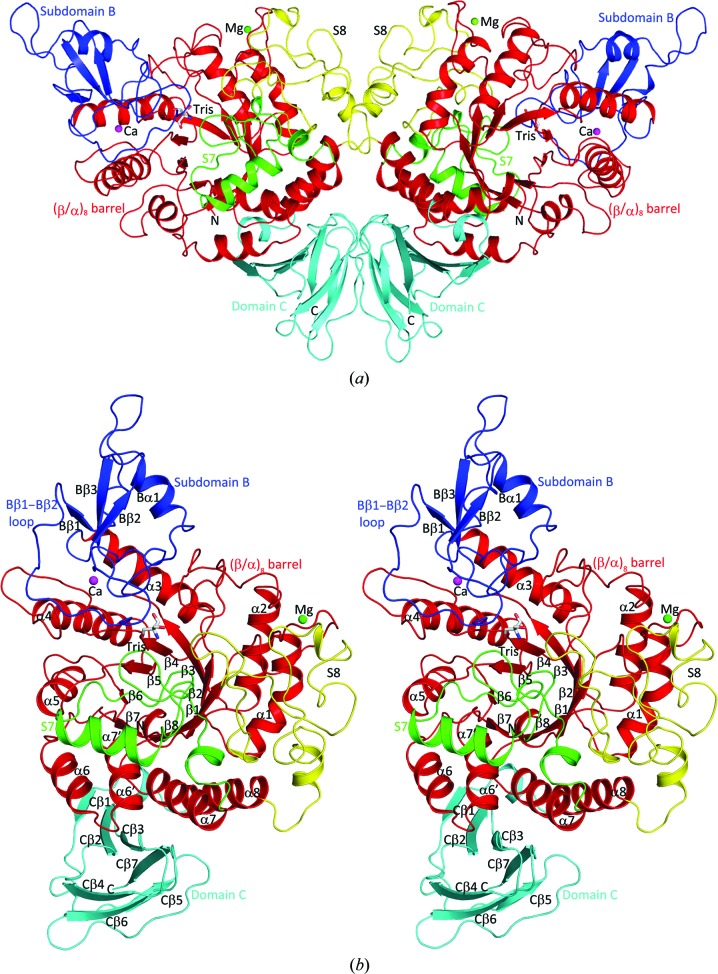
Ribbon views of the DrTS dimer (*a*) and monomer (*b*) structures. The protein consists of a (β/α)_8_ barrel (red), subdomain B (blue), domain C (cyan) and two TS-unique modules (S7 in green and S8 in yellow). The bound Mg^2+^ and Ca^2+^ ions are shown as green and magenta spheres, respectively, with the Tris molecule shown as a stick representation. The dimeric interfaces are formed mainly by domain C and S8 and have a total buried area of ∼3000 Å^2^.

Analytical ultracentrifugation experiments were able to clearly demonstrate that DrTS exists as a dimer in solution as well as in the crystal form, with each asymmetric unit containing two and four DrTS dimers in space groups *P*2_1_2_1_2_1_ and *P*2_1_, respectively (Figs. 1 ▶ and 2 ▶ *a*). Clear electron density was observed covering the entire DrTS molecule, except for residues 1–5 and the vector linkers. The four or eight subunits in each asymmetric unit did not display significant differences, with root-mean-square deviations of 0.3–0.4 Å for all C^α^ atoms. As in other GH13 members, each DrTS protomer mainly consists of a catalytic (β/α)_8_ barrel (β1–β8, α1–α8 and α6′) as the central core and a C-terminal β-sandwich (residues 463–552; domain C) (Fig. 2 ▶ *b*). Domain C is made up of two antiparallel β-sheets with five (Cβ1–Cβ3, Cβ5 and Cβ7) and two (Cβ4 and Cβ6) β-strands. This region is tightly connected to the (β/α)_8_ barrel through an extensive network of inter­actions consisting of nine hydrogen bonds and various hydrophobic patches involving 47 residues, which are mainly contributed by its five-stranded β-sheet and α6, α6′, α7 and α8. In TSs, domain C has the greatest diverse in sequence; while DrTS shares ∼50% sequence identity with MtTS overall, they display only ∼25% sequence identity in domain C.

Three additional subdomains are formed by long extrusion fragments inserted between β3 and α3, between β7 and α7, and between β8 and α8 within the (β/α)_8_ barrel, and they are designated as sub­domains B, S7 and S8, respectively (Fig. 2 ▶ *b*). These sub­domains are all loop-rich structures. Subdomain B (residues 105–184) is common to the GH13 family and consists of one short helix (Bα1) and three antiparallel β-strands (Bβ1–Bβ3). This subdomain strongly interacts with the (β/α)_8_ barrel by forming 16 hydrogen bonds and extensive hydrophobic contacts between 58 residues. On the other hand, subdomains S7 and S8 are unique to TS. Specifically, S7 (residues 315–361) contains a short helix (α7′), while S8 (residues 384–449) does not display any significant secondary structure. The (β/α)_8_ barrel contains the catalytic cleft, which consists of the triad Asp209–Glu251–Asp319 and various conserved substrate-binding residues. Domain C and sub­domain S8 contribute to dimer formation, while subdomains B and S7 serve as a gateway for the opening and closing of the active site. These points will be discussed later.

In addition, many GH13 members contain metal ions, which may be involved in the maintenance of structural integrity (Kobayashi *et al.*, 2011 ▶). In the α-amylase from the human gut bacterium *Bacteroides thetaiotaomicron* (BtSusG) and MsTS, one Mg^2+^ and one Ca^2+^ ion were identified (Koropatkin & Smith, 2010 ▶; Caner *et al.*, 2013 ▶). In DrTS two similar metal ion-binding sites can also be observed and hence the same divalent cations have been modelled into the electron density (Fig. 2 ▶ *b*). The Mg^2+^ site consists of the side chains of Asp24, Asn26, Asp28 and Asp32 and the main-chain O atom of Lys30, which form part of the consensus signature D*X*N*X*DG*X*GD. Such a site is also found in some other GH13 members, for instance the similar calcium sites in *Rhizobium* sp. MX-45 sucrose isomerase (trehalulose synthase; RhSI) and in yeast iso­maltase (ScIM; Ravaud *et al.*, 2007 ▶; Yamamoto *et al.*, 2010 ▶). On the other hand, the Ca^2+^ site is coordinated by the side chains of Asn105, Asp179 and Glu216 and the backbone O atoms of Tyr213 and Leu214. Many α-amylases contain a similar calcium site that involves the conserved asparagine and aspartate residues (Asn105 and Asp179 in DrTS; Janeček *et al.*, 2014 ▶). Interestingly, in the closest structural neighbours of TS this site is occupied by a lysine, which replaces the ligand glutamate residue (Glu216 in DrTS).

### 3.2. Closest structural neighbours   

**Figure 3:**
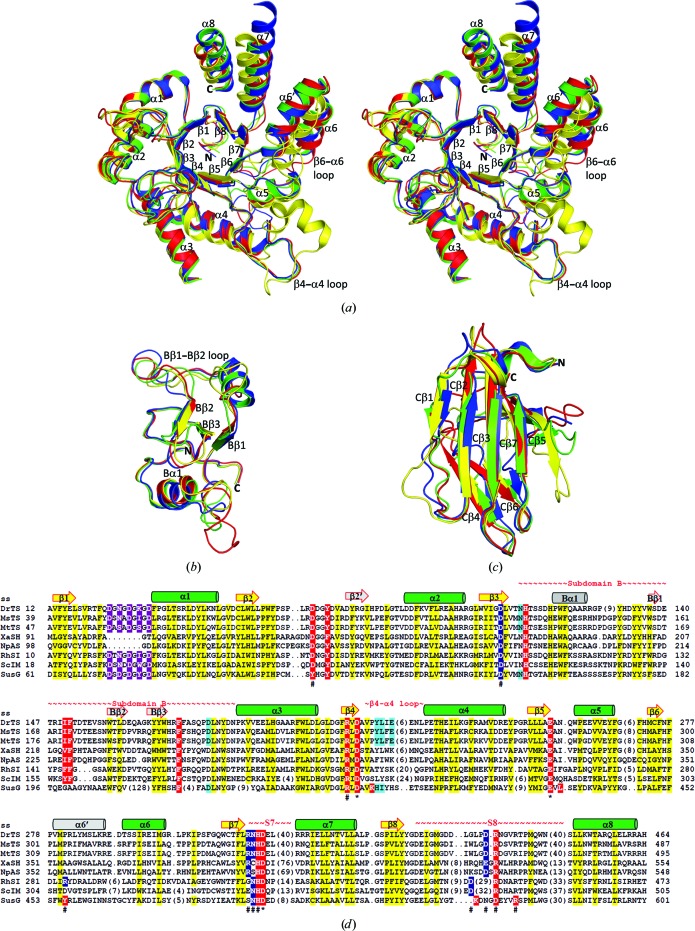
Closest structural neighbours of DrTS. Structural superpositions of DrTS-N253A–Tris (red), XaSH-E322Q–sucrose (blue; PDB entry 3czk), NpAS-E328Q–maltoheptaose (green; PDB entry 1mw0) and RhSI-E254Q–sucrose (yellow; PDB entry 2pwe) for the (β/α)_8_ barrel (*a*), subdomain B (*b*) and domain C (*c*). These enzymes share a conserved (β/α)_8_ barrel, parts of subdomain B and a structurally conserved five-stranded sheet in domain C, particularly the Cβ2, Cβ3 and Cβ7 strands. (*d*) Structure-based sequence alignment of DrTS and its close homologues. Secondary-structure elements of DrTS are labelled and the numbers of residues in gaps are indicated in parentheses. BtSusG contains an additional carbohydrate-binding module inserted between Bβ2 and Bβ3. The residues of the Mg^2+^ and Ca^2+^ sites are shaded in magenta and cyan, respectively. The substrate-binding residues are shaded in red, whereas the residues of the conserved hydrophobic core are shaded in yellow. The catalytic triad residues are indicated with an asterisk, while the residues involved in stabilization of the triad orientation, but not in substrate binding, are shaded in blue and indicated with a hash, whereas those involved in both functions are shaded in red and indicated with a hash.

A search using the *DALI* server (Holm & Rosenström, 2010 ▶) revealed that DrTS shares the greatest amount of structural similarity with MsTS, MtTS, *Xanthomonas axonopodis* sucrose hydrolase (XaSH), *Neisseria polysaccharea* amylosucrase (NpAS), RhSI, ScIM and BtSusG, with root-mean-square deviations (r.m.s.d.s) of 1.8 Å (PDB entry 3zoa; 530 C^α^ atoms with 52% sequence identity), 2.1 Å (PDB entry 4lxf; 524 C^α^ atoms with 53% identity), 1.7 Å (PDB entry 3czk; 482 C^α^ atoms with 28% identity), 1.9 Å (PDB entry 1mw0; 485 C^α^ atoms with 28% identity), 2.3 Å (PDB entry 2pwe; 476 C^α^ atoms with 33% identity), 2.4 Å (PDB entry 3axh; 476 C^α^ atoms with 29% identity) and 2.6 Å (PDB entry 3k8l; 458 C^α^ atoms with 31% identity), respectively (Caner *et al.*, 2013 ▶; Roy *et al.*, 2013 ▶; Kim *et al.*, 2008 ▶; Skov *et al.*, 2002 ▶; Ravaud *et al.*, 2007 ▶; Yamamoto *et al.*, 2011 ▶; Koropatkin & Smith, 2010 ▶; Fig. 3 ▶). The (β/α)_8_ barrel is the most superimposable part of the structure, as are parts of subdomain B and domain C (Figs. 3 ▶ *a*, 3 ▶ *b* and 3 ▶ *c*). The most divergent parts of the (β/α)_8_ barrel are the member-unique modules inserted between β4 and α4, between β6 and α6, between β7 and α7 (S7), and between β8 and α8 (S8) (Figs. 3 ▶ *a* and 3 ▶ *d*), which are located on the top of the active site and may modulate the size/shape and accessibility of the active site. In subdomain B, the most diverse segment is the region between Bβ1 and Bβ2, the so-called Bβ1–Bβ2 loop (Fig. 3 ▶ *b*), which lines one side of the active-site entrance and hence is involved in substrate access and binding. The member-unique insertions and subdomain B will be discussed later.

Domain C displays a distinct lack of significant sequence similarity. Superposition of domain C reveals that the five-stranded β-sheet is more superimposable, whereas the two-stranded β-sheet is more diverse (Fig. 3 ▶ *c*). The former is tightly packed with the (β/α)_8_ barrel, while the latter may contribute to the functional diversity. For example, in TS this two-stranded β-sheet makes up the dimer interface, while in a number of GH13 members this part may function as a carbohydrate-binding module (Christiansen *et al.*, 2009 ▶). In contrast to most GH13 members, which are monomeric, DrTS is dimeric, while MtTS forms a tetramer (Roy *et al.*, 2013 ▶). The dimeric interfaces are mainly mediated by the TS-unique S8 and domain C (Fig. 2 ▶ *a*). Owing to the sequence diversity of domain C, the hydrogen bonds and hydrophobic contacts in the dimeric interfaces of DrTS and MtTS are distinct. This difference accounts for the various oligomer states. In DrTS, there are 26 direct hydrogen bonds between the protein atoms across the dimer interface, whereas there are only ten direct hydrogen bonds in MtTS. As a result, dimeric MtTS may not be stable and a tetramer is thus formed with seven direct hydrogen bonds and ten salt bridges through subdomains B and S8. Oligomerization of the TS may enhance the structural stability, particularly in the loop-rich S8 and the two-stranded β-sheet of domain C. In monomeric GH13 members such as XaSH, NpAS and RhSI, the two β-strands are more flexible and much shorter than those in TS (Fig. 3 ▶ *c*), and are even absent in ScIM and sucrose phosphorylase (Yamamoto *et al.*, 2010 ▶; Sprogøe *et al.*, 2004 ▶). To explore the dimer function, the generation of monomeric DrTS mutants is being performed and their activity will be investigated.

### 3.3. The similar active-site architecture   

**Figure 4:**
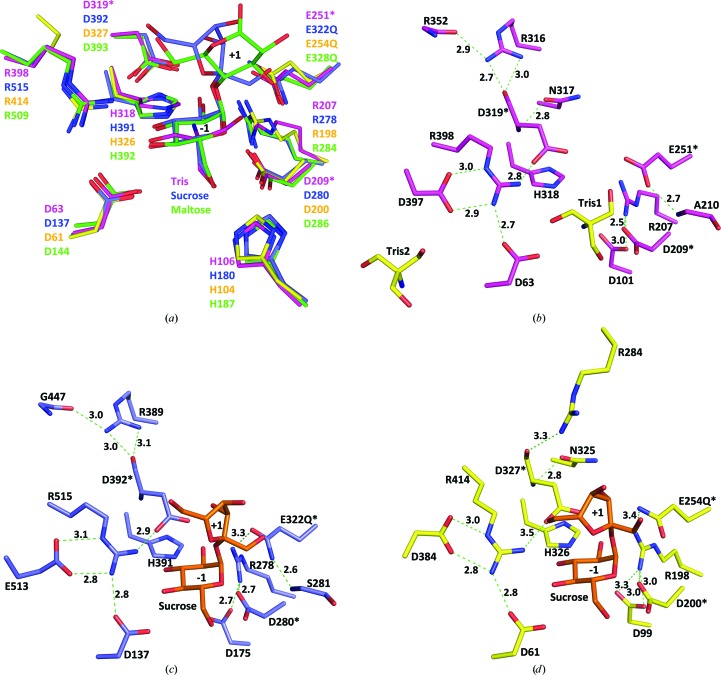
The similar active-site architecture. (*a*) Superposition of the −1 subsites of DrTS-N253A–Tris (megenta), XaSH-E322Q–sucrose (slate), NpAS-E328Q–maltoheptaose (green) and RhSI-E254Q–sucrose (yellow). The residue numbering is labelled in the same colour for each protein. The conserved active-site residues of DrTS occupy similar spatial positions to those of XaSH, NpAS and RhSI. Interestingly, the nonreducing terminal maltosyl residue in the NpAS–maltoheptaose complex fits the −1 and +1 subsites of DrTS well. (*b*)–(*d*) The interaction networks between the catalytic triad and the surrounding residues in DrTS-N253A–Tris (*b*), XaSH-E322Q–sucrose (*c*) and RhSI-E254Q–sucrose (*d*). The triad residues are indicated by an asterisk.

The available crystal structures of GH13 enzymes in complex with substrates (or substrate analogues) display conserved active-site residues that form virtually identical interaction networks with the glucose residue at the −1 subsite (Kim *et al.*, 2008 ▶; Skov *et al.*, 2002 ▶; Ravaud *et al.*, 2007 ▶; Yamamoto *et al.*, 2011 ▶). These extensive interactions not only result in tight binding but also define the orientation of the nonreducing end to the −1 subsite for the double-displacement catalytic mechanism. A detailed structural comparison revealed that the active-site architecture of our DrTS structure resembles those of many other GH13 members such as its close structural matches (Fig. 4 ▶). The conserved active-site residues of DrTS occupy similar spatial positions and hence may form nearly identical interactions with the glucose residue at the −1 subsite upon substrate binding (Fig. 4 ▶ *a*).

In addition, our comparison revealed that the conserved active-site residues form similar extensive interaction networks with the catalytic triad (Figs. 4 ▶ *b*, 4 ▶ *c* and 4 ▶ *d*). Such interaction networks may be responsible for the attainment of the active conformation, as discussed below. In DrTS, the nucleophile Asp209 forms a salt bridge with Arg207, which also forms a salt bridge with Asp101, while the general acid/base Glu251 forms a hydrogen bond to the carbonyl backbone of Ala210 (Fig. 4 ▶ *b*). Interestingly, the transition-state stabilizer Asp319 forms a more extensive network with its surrounding residues. The carboxylate side chain of Asp319 stacks with His318 and forms a salt bridge with Arg398, which also forms salt bridges with Asp63 and Asp397. In addition, the carbonyl backbone of Asp319 forms a hydrogen bond to Arg316, which also hydrogen-bonds to the carbonyl backbone of Arg352. Virtually identical interaction networks are observed in XaSH and NpAS (Fig. 4 ▶ *c*). Interestingly, structurally homologous but not sequence-equivalent Arg and Asp residues have been found to sustain the transition-state stabilizer Asp. For example, in RhSI the corresponding residue to Arg316 in DrTS is Gly324, and RhSI instead utilizes the first arginine (Arg284) in its unique isomerization motif to interact with the transition-state stabilizer Asp327 (Fig. 4 ▶ *d*; Ravaud *et al.*, 2007 ▶). Disruption of the interactions surrounding Asp327 in the randomly mutated R284C variant with major hydrolytic activity causes a considerable change in subdomain orientations, leading to an open conformation (Lipski *et al.*, 2013 ▶). In addition, the corresponding pair to Asp63–Arg398 in DrTS is absent in endo-α-amylases, which instead use another conserved Asp–Arg pair (Asp545 and Arg549 in BtSusG) for both substrate binding and active-site stability (Fig. 3 ▶ *d*; Koropatkin & Smith, 2010 ▶; Janeček *et al.*, 2014 ▶).

### 3.4. The substrate-binding site   

**Figure 5:**
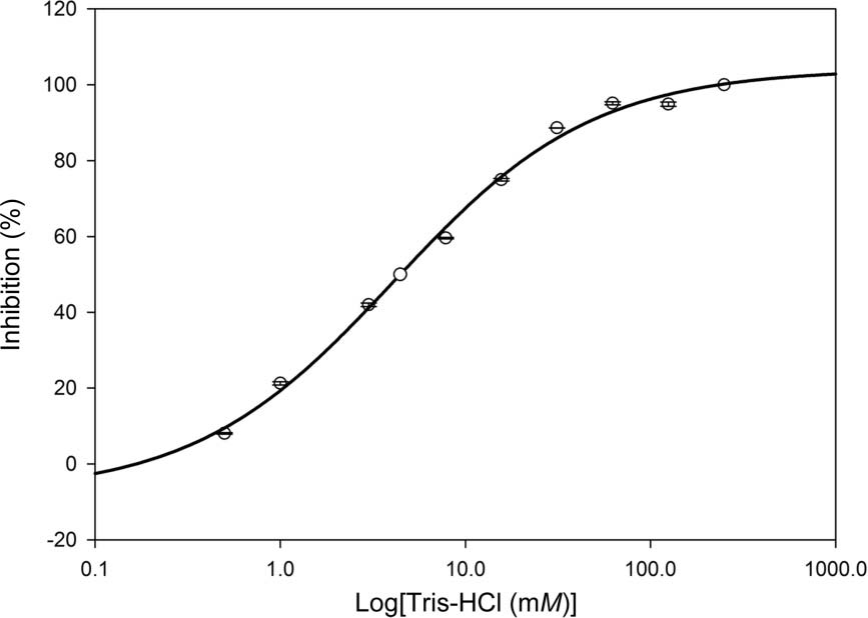
The inhibitory effect of Tris on the isomerase activity of DrTS. An IC_50_ value of 4.88 m*M* was estimated. All experiments were carried out in duplicate or triplicate.

**Figure 6:**
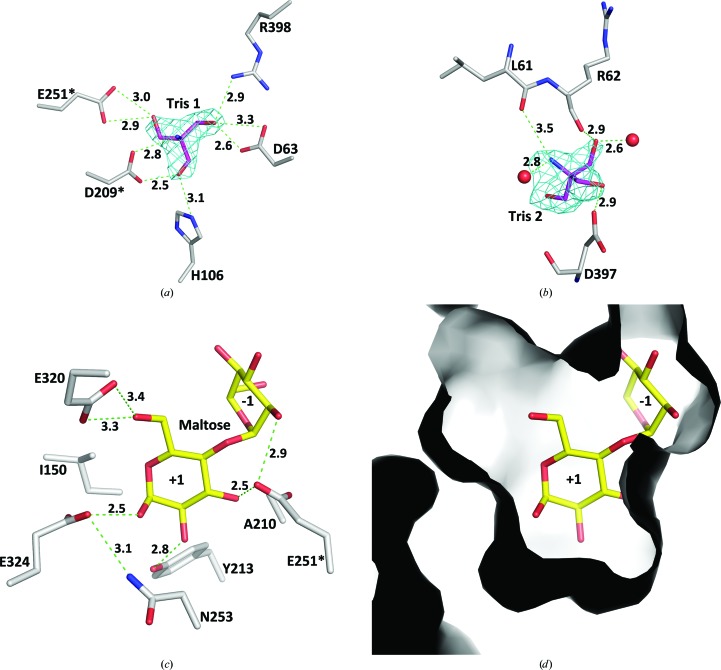
The substrate-binding site in DrTS. (*a*) The Tris bound at the −1 subsite in the N253A mutant. The simulated-annealing *F*_o_ − *F*_c_ OMIT map for Tris is contoured at the 6σ level. (*b*) An additional Tris-binding site in the N253A variant. The simulated-annealing *F*_o_ − *F*_c_ OMIT map is contoured at the 4σ level. (*c*) The interaction networks between DrTS and the modelled maltose in the +1 subsite. Hydrogen bonds are shown as green dashed lines. (*d*) Molecular surfaces of the +1 subsite. The view is the same as that in (*c*).

In a number of GH13 enzymes, Tris has been shown to be a competitive inhibitor and is able to mimic a sugar molecule bound at the −1 subsite (Skov *et al.*, 2002 ▶; Ravaud *et al.*, 2007 ▶). An inhibitory effect of Tris on the activity of TS has also been observed (Filipkowski *et al.*, 2012 ▶; Jiang *et al.*, 2013 ▶). Our present assay results indicated that the activity of DrTS is inhibited strongly by Tris, with a IC_50_ value of 4.88 m*M* in the presence of 50 m*M* maltose (Fig. 5 ▶). In the N253A structure at 2.21 Å resolution, a strong electron-density peak was observed at the −1 subsite that correlates well with the shape of a Tris molecule (Fig. 6 ▶ *a*). The peak was assigned as a Tris from the crystallization solution, which contained 300 m*M* Tris–HCl, forming hydrogen bonds to Asp63, His106, Asp209, Glu251 and Arg398. In the wild-type structure, a similar Tris signal was also observed. In the N253A mutant, an additional Tris-binding site was detected near to the −1 subsite, and the Tris molecule forms hydrogen bonds to two water molecules, the carboxylate group of Asp329 and the backbones of Leu61 and Arg62 (Fig. 6 ▶ *b*).

Interestingly, in addition to the nearly identical active-site architecture of DrTS to those of XaSH and NpAS, the nonreducing terminal maltosyl residue in the NpAS–maltoheptaose complex fits the −1 and +1 subsites in DrTS–Tris well (Fig. 4 ▶ *a*), and hence a maltose was modelled into the active site (Figs. 6 ▶ *c* and 6 ▶ *d*). After modelling, energy minimization was performed in *CNS* as a structural refinement (Brünger *et al.*, 1998 ▶). The modelled DrTS–maltose structure suggested that Ile150, Ala210, Tyr213, Glu320 and Glu324 are involved in the +1 subsite (Fig. 6 ▶ *c*). A conserved phenyl­alanine is present in the sucrose isomerases and therefore the I150F variant was constructed. I150F was found to display ∼55% of the isomerase activity and ∼170% of the hydrolase activity of the wild type. Thus, at this spatial position in DrTS an isoleucine is more favourable for maltose isomerization rather than a phenylalanine. However, neither isomerase nor hydrolase activity was detected when the Y213A, E320A and E324A mutants were examined, which indicates that these residues are essential for TS activity.

### 3.5. Substrate-induced conformational changes   

**Figure 7:**
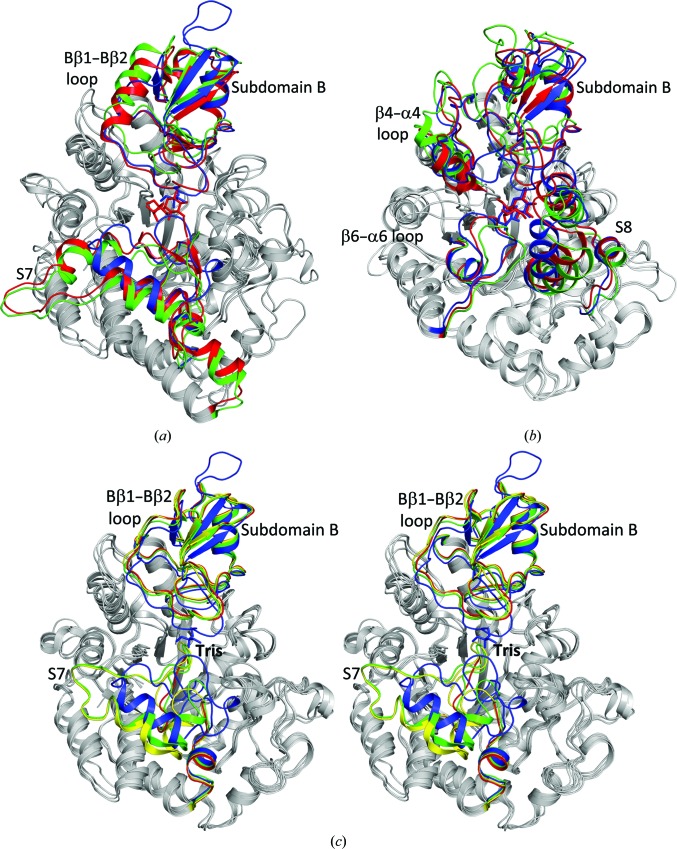
Different conformations observed in SH, SI and TS. Structural superposition of (*a*) XaSH-E322Q–sucrose (red), apo XcSH (green; PDB entry 2wpg) and DrTS–Tris (blue) and of (*b*) RhSI-E254Q–sucrose (red), the RhSI R284C mutant (green; PDB entry 4h2c) and ScIM-E277A–isomaltose (blue; PDB entry 3axh). Some of the member-unique insertions such as subdomains B and S7 in TS, SH and AS, or subdomains B and S and the segments between β4 and α4 and between β6 and α6 in SI and ScIM, modulate the size/shape and accessibility of the active site. These modules may move forward and backward from the active site to form different conformations during enzyme catalysis. (*c*) Stereoview of the structural superposition of DrTS–Tris, MsTS (PDB entry 3zoa) and MtTS (PDB entry 4lxf) chain *A* and chain *B*. The subdomain B and S7 are shaded in blue, green, yellow, and red, respectively. These display distinct tertiary structures of S7 and the relative orientations of the subdomain B to the (β/α)_8_ barrel are different. A similar rearrangement of subdomain B was observed in TS, AS and SH.

The available crystal structures of SH, AS, SI and ScIM reveal that upon substrate binding the member-unique modules inserted within the (β/α)_8_ barrel move forward to cover and even to seal the active-site entrance (Fig. 7 ▶). A structural comparison of XaSH–sucrose and the apo *X. campestris* SH (XcSH) suggested that upon sucrose binding the Bβ1–Bβ2 loop becomes ordered and subdomains B and S7 move forward to form the active conformation (Fig. 7 ▶ *a*; Kim *et al.*, 2008 ▶; Champion *et al.*, 2009 ▶). In addition, the structures of NpAS, with 36% sequence identity to XaSH, display a similar conformation when in complex with Tris, glucose, sucrose and maltoheptaose (Skov *et al.*, 2002 ▶). However, the apo *D. radiodurans* AS (DrAS) showed a strikingly different conformation in which the β2–α2 loop is disordered and subdomains B and S7 swing away to create a more open active site (Skov *et al.*, 2013 ▶). Moreover, the RhSI structures show a similar closed conformation when in complex with Tris, glucose, sucrose and inhibitors (Ravaud *et al.*, 2007 ▶), whereas the R284C variant displays an open conformation with a drastic rearrangement of the SI-unique inserted modules, particularly subdomain B and the isomerization loop between β6 and α6 (Fig. 7 ▶ *b*; Lipski *et al.*, 2013 ▶). The available ScIM structures display an almost closed conformation and the mechanism of substrate binding to the active site remains unclear (Yamamoto *et al.*, 2010 ▶, 2011 ▶). Since ScIM shares 38% sequence identity and high structural homology with RhSI, it is possible that subdomain B and its unique modules, particularly the β4–α4 and β6–α6 loops, may be involved in the opening and closing of the active site (Fig. 7 ▶ *b*).

The crystal structures of DrTS, MsTS and MtTS display two significant structural differences (Fig. 7 ▶ *c*). Firstly, the relative orientations between (sub)domains are different; in particular, subdomain B in MsTS and MtTS rotates away from the active site by ∼9°. Secondly, the tertiary structures of sub­domain S7 are markedly distinct even if there is >80% sequence identity between them. Interestingly, a similar rotation of subdomain B by ∼9° and changes in S7 were also observed when substrate binds to SH and AS as described above. The structures of the the XaS–sucrose, NpAS–maltoheptaose and our DrTS–Tris complexes share a similar relative orientation of subdomain B, particularly a similar spatial position of the Bβ1–Bβ2 loop (Fig. 7 ▶ *a*), while those of apo XcSH, apo DrAS, MsTS and MtTS also display a similar subdomain B orientation. Because of high structural homology, in particular similar active-site architectures and subdomain B orientations of TS to SH, AS and SI (Figs. 3 ▶, 4 ▶ and 7 ▶), we therefore conclude that the MsTS and MtTS structures may represent an open conformation of apo TS, while our DrTS–Tris complex represents a substrate-induced closed conformation for catalysis of the intramolecular isomerization.

### 3.6. Open and closed TS conformations   

**Figure 8:**
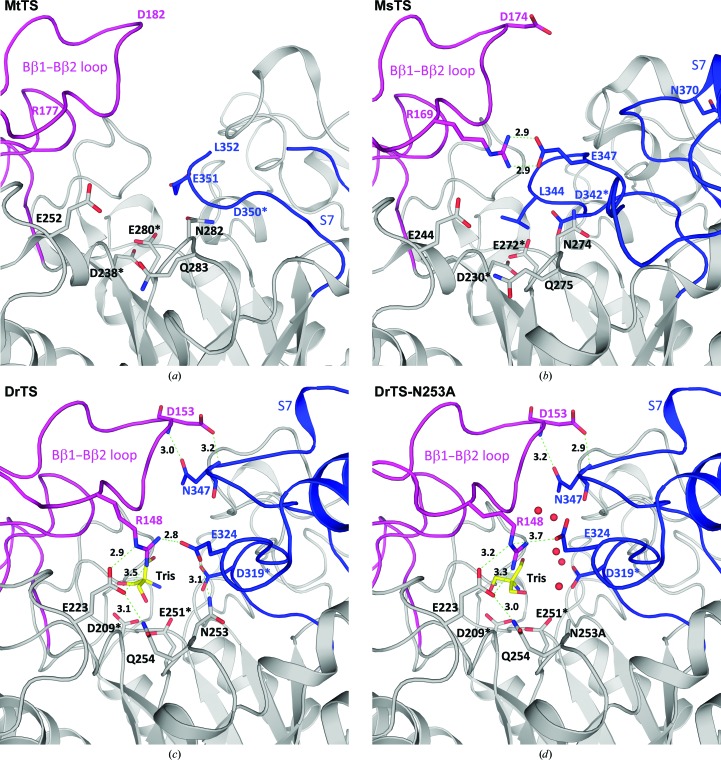
The interaction networks between subdomains B (magenta) and S7 (blue) in MtTS (PDB entry 4lxf; chain *B*) (*a*), MsTS (*b*), DrTS–Tris (*c*) and DrTS-N253A–Tris (*d*). In one MtTS structure the active site is wide open owing to a disordered region in S7, while in MsTS Leu344 blocks the substrate-binding pocket. On the other hand, in DrTS a closed conformation was observed. These TS structures indicate that subdomains B and S7 serve as a gateway for the opening and closing of the active site. In N253A, the absence of the hydrogen bond between Asn253 and Glu324 causes movement of the Glu324 side chain, leading to the creation of a small pore for water entry.

The crystal structure of MtTS showed two conformations (Roy *et al.*, 2013 ▶). In one conformation, residues 353–381 in S7 are disordered and the active site is wide open to solvent (Figs. 7 ▶ *c* and 8 ▶ *a*). On the other hand, the other MtTS structure is nearly identical to the MsTS structure (Fig. 7 ▶ *c*). S7 is composed of two short α-helices along with an extended loop, and the transition-state stabilizer Asp was shifted away from the active site by ∼4 Å (Caner *et al.*, 2013 ▶; Roy *et al.*, 2013 ▶). In addition, Leu344 in MsTS, which is Leu352 in MtTS, projects into the active site and blocks the substrate-binding pocket (Fig. 8 ▶ *b*). Compared with some GH13 members (Fig. 4 ▶), the active-site residues in the MsTS and MtTS structures adopt different spatial positions, and in addition the transition-state stabilizer Asp does not interact with its surrounding residues.

In our DrTS–Tris complex, the interaction networks formed between subdomains B, S7 and the active-site residues seal the active-site entrance (Fig. 8 ▶ *c*). In particular, Arg148 forms salt bridges with Glu223 and Glu324, which form hydrogen bonds to Gln254 and Asn253, respectively. In addition, Asp153 forms two hydrogen bonds to Asn347. In DrTS, Ile150, Phe151 and Phe173 in subdomain B and His318, Asp319, Glu320 and Glu324 in S7 all constitute part of the active-site pocket. This means that interactions of the substrate with these residues would bring these two subdomains closer together to form a closed conformation. Such an enclosed active site in TS is needed for catalysis of the intramolecular isomerization reaction while minimizing hydrolysis of the sugar. The crystal structures of the wild type and the N253A mutant are nearly identical, except for the side-chain orientation of Glu324 (Fig. 8 ▶ *d*). In the N253A mutant, disruption of the interaction between Asn253 and Glu324 results in movement of the Glu324 side chain to create a small pore for solvent entry to the active site. This may explain why the N253A mutant has ∼11% of the isomerase activity and ∼180% of the hydrolase activity of the wild type. Similar to N253A, the R148A mutant shows ∼12% of the isomerase activity and ∼150% of the hydrolase activity of the wild type, suggesting that a small pore for water entry may also be present when Arg148 is replaced by alanine.

### 3.7. Implications into the enzymatic mechanisms of TS   

Mechanistic analysis of MsTS demonstrated that the TS-catalyzed isomerization of maltose to trehalose employs a two-step double-displacement mechanism (Zhang *et al.*, 2011 ▶). The substrate binding allows subdomains B and S7 to move toward and seal the active-site pocket (Fig. 8 ▶ *c*). Kinetic studies of MsTS suggested that such closure of the active site is the rate-limiting step. The nucleophile Asp attacks the anomeric centre of the glucose at the −1 subsite, yielding the covalent glycosyl-enzyme intermediate. The narrow and enclosed active site in DrTS and RhSI not only protects the glucosyl-enzyme intermediate from water, thus avoiding hydrolysis, but also allows the cleaved glucose/fructose at the +1 subsite to be retained to reorient and finally to re-attack the covalent intermediate in order to complete the isomerization reaction intramolecularly. Our present studies on DrTS will help to provide additional information that should aid in the modification of this enzyme for potential use in industrial applications.

## Supplementary Material

PDB reference: DrTS, wild type, 4tvu

PDB reference: N253A mutant, 4wf7

## Figures and Tables

**Table 1:** Statistics of data collection and structural refinement Values in parentheses are for the highest resolution shell.

	Wild type	N253A
PDB code	4tvu	4wf7
Data collection		
Space group	*P*2_1_	*P*2_1_2_1_2_1_
Unit-cell parameters (, )	*a* = 95.61, *b* = 195.90, *c* = 130.99, = = 90, = 92.89	*a* = 96.25, *b* = 133.71, *c* = 196.61, = = = 90
Resolution ()	302.70 (2.802.70)	202.21 (2.292.21)
Observed reflections	462831 (38844)	759037 (71092)
Unique reflections	128557 (11771)	129348 (12695)
Completeness (%)	97.7 (89.4)	99.9 (99.7)
Multiplicity	3.6 (3.3)	5.9 (5.6)
*I*/**(*I*)	13.1 (2.9)	12.6 (2.3)
*R*_merge_ † (%)	9.6 (40.8)	15.1 (78.7)
Refinement		
Resolution ()	302.7 (2.772.70)	202.21 (2.272.21)
Reflections [*F*> 0(*F*)]	122065 (8113)	119863 (8156)
*R*_cryst_ ‡ (%)	19.4 (28.2)	16.0 (22.6)
*R*_free_ § (%)	23.6 (31.9)	20.0 (26.3)
R.m.s. deviations		
Bond lengths ()	0.01	0.01
Bond angles ()	1.42	1.41
Mean *B* value (^2^)		
Protein	38.0	33.0
Metal ions	27.1	26.7
Tris	32.5	35.9
Water	24.9	37.3
Ramachandran analysis¶ (%)		
Favoured	97.4	97.4
Allowed	2.6	2.6
Disallowed	0	0

† *R*_merge_ = 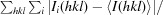 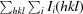, where *I_i_*(*hkl*) is the average intensity value of the equivalent reflections.

‡ *R*_cryst_ = 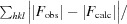 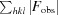, where *F*_obs_ and *F*_calc_ are the observed and the calculated structure factors, respectively.

§ *R*_free_ was calculated using 5% of data that were randomly excluded from refinement.

¶ The Ramachandran analysis was performed by *MolProbity* (Chen *et al.*, 2010 ▶).
